# From engagement to competency: The pathway to making disability naïve frontline workers competent in the delivery of an evidence-based autism intervention in New Delhi, India

**DOI:** 10.3389/fpsyt.2022.903341

**Published:** 2022-07-29

**Authors:** Lavangi Naithani, Priya Sangwan, Sanjana Guha Roy, Sreepriya Menon, Zakiya Azar, Shweta Lakhera, Divya Kumar, Minal Kakra Abhilashi, Reetabrata Roy, Vivek Vajaratkar, Carol Taylor, Vikram Patel, Jonathan Green, Gauri Divan

**Affiliations:** ^1^Sangath, New Delhi, India; ^2^Division of Neuroscience and Experimental Psychology, University of Manchester, Manchester, United Kingdom; ^3^Harvard Medical School, Boston, MA, United States

**Keywords:** Accredited Social Health Activist (ASHA), autism spectrum disorders (ASD), evidence-based intervention, engagement, recruitment, competency, non-specialist health workers

## Abstract

**Background:**

As countries like India improve access to maternal and infant care, the health systems need to develop services that enable all children to thrive. A key demographic which needs to be supported are children with disabilities, such as autism. With an estimated prevalence of one percent, there are over five million young children who need services to support their needs. However, the paucity of specialist care makes access to interventions difficult. In this context a public health research not-for-profit is evaluating the effectiveness of the task-sharing approach to support the delivery of an evidenced social communication intervention for young children with autism. This paper describes the process of engaging and training the non-specialist frontline Accredited Social Health Activists (ASHAs), who are embedded within the Ministry of Health and Family Welfare under the Delhi State Health Mission, to deliver a complex intervention for autism to inform the future scalability of services for neurodevelopmental disorders.

**Methods:**

The present study describes the process which included (i) engagement meetings, (ii) recruitment, (iii) training, (iv) internship, and (v) competency evaluation. The shortlisted ASHAs received a 7-day classroom training followed by an internship period with practice cases. Finally, competency assessments, comprising of a test of knowledge and skills through role-plays, was administered.

**Results:**

Twenty three Primary Urban Health Centers across seven districts of Delhi were approached and 408 ASHAs were engaged in initial meetings. Telephonic screening with 127 ASHAs resulted in 72 ASHAs being selected for in-person interviews. Of the 45 ASHAs who attended, 33 were shortlisted for training and 18 completed it. Fifteen ASHAs entered the internship of which 7 ASHAs achieved competency.

**Discussion and conclusion:**

There was significant attrition along the pathway to having a competent non-specialist worker deliver a complex autism intervention. The lessons learnt from this process can inform the possibility of developing a cadre of disability specific frontline health workers who can deliver evidence-based interventions for neurodevelopmental disorders under supervision.

## Background

India has unique challenges in providing universal health and rehabilitation coverage to its population of 1.3 billion. Though improvements in the provision of health care over the last 75 years has had a positive impact on life expectancy, non-communicable diseases (which include mental health and neurological disorders) contribute a significant burden to death and disabilities (1). Individuals with Autism Spectrum Disorders or autism [hereafter] have a core social communication impairment, restricted and repetitive sensory—motor behaviors. It is a pervasive lifelong condition, where families and individuals require long term support which varies as the individuals' needs change over time (2). However, evidence-based early interventions can transform the trajectory and support increased inclusion and participation of an individual (3, 4). A population-based study of neurodevelopmental disorders in children aged 2–9 years in India, revealed that one in eight children have a neurodevelopmental disability including hearing impairment and intellectual disability (5). Amongst these, autism has an estimated prevalence of 1.12 (0.74–1.68) per 100 children (5). Unfortunately, the majority of these children in India do not receive evidence-based interventions and families struggle to access appropriate care (6).

Even in 2017, the country's doctor to patient ratio was less than the WHO's minimum recommendation of 1 doctor for every 1,000 people of the population (7). This acute lack of human resources was addressed by the Ministry of Health and Family Welfare (MoHFW) by establishing the National Rural Health Mission in 2005 and introducing the Accredited Social Health Activist (ASHA). This cadre of frontline health workers was designated with the task of promoting health awareness at a community level with an aim to increasing the utilization of primary health services by mobilizing populations (8, 9), and providing services for maternal and child healthcare (10).

The mission now deploys ASHAs across 32 states and Union territories in India, and an individual ASHA on an average caters to a population of 1,000 individuals (11). Their required characteristics include being women between the ages of 20–45 years, residents in their communities of work, who are or were married (12) with a minimum eight grade education (13). On recruitment, they receive foundational modular training of up to 5 weeks (14) on a range of topics which includes community health and rights, maternal health, new-born care, infant and young child nutrition, reproductive health and infections (15). The ASHA's role is incentivized against monthly targets which are contextually set, based on the needs of their communities. Studies have observed that the health outcomes of communities are reflected more by the nature of training (content and duration) provided to ASHAs, as against their educational qualifications (10). The importance of regular refreshers (13, 16) and competency-based trainings along with mentorship for monitoring knowledge levels while maintaining the skills and motivation of ASHAs (10) has also been observed.

The establishing of the Health Mission along with other policy moves have resulted in improvements in health statistics for example, a decline in infant mortality from 74.4 deaths per 1,000 live births in 2005 to 32 deaths per 1,000 in 2018 (17), and a shift in focus from child survival to supporting children to thrive. With this in mind the MoHFW launched the Rashtriya Bal Swasthya Karyakram (RBSK) in 2013, with an aim of providing early identification and interventions for children (0–18 years). The RBSK program covers 30 conditions including developmental delays and disabilities. Its services are delivered through the District Early Intervention Centres (DEIC) which are designated for interventions and follow ups, including the provision of specialized therapies for autism (11). The ASHAs role in it is to support home-based screening and monitoring of children. Though in theory, the RBSK aims to provide comprehensive diagnostic and rehabilitative services, studies have pointed out the manpower shortage in the DEICs (18–20), a high turnover of staff (20) which impacts the care being delivered and an uneven roll out across the states of India. As a result, most services for children with disabilities including autism are only available in metropolitan centers which require long commutes to specialized facilities, with associated intervention and transportation cost and loss of daily wages for many families (21, 22). Within this context, a not-for-profit research organization, Sangath adapted an evidence-based intervention for autism to be delivered in the community using the process of task-sharing (22). Task-sharing is a successful strategy in addressing the paucity of specialists in low resource settings by allowing specialists to transfer certain skilled roles to non-specialists under supervision (23).

PASS, a social communication intervention for children with autism; has been adapted and expanded with a “Plus” component for task-sharing with non-specialists from the UK developed PACT intervention (24, 25). The non-specialists are supported with training and supervision to deliver 12 sessions to each parent—child dyad every fortnight over a period of 8 months. The intervention uses a 6–8-min parent-child play session recorded live on a smartphone. This is used by the counselor to guide the parent's learning of strategies which support social communication through reflective video feedback on segments of the recorded play. The parents are encouraged to practice the strategies for half an hour every day with their child to help reinforce new ways of interacting with their child. The Plus component supports the family with strategies for commonly co-occurring problems. Two pilot randomized controlled trials in South Asia set in two states of India, Goa and Kolhapur, have evaluated the acceptability and feasibility of the non-specialist health worker delivery of this intervention for autism (24, 26). Both trials used project-based non-specialists. However, to answer the question on scalability and sustainability, the aim of this current study is to determine whether an existing frontline worker; the ASHA could be trained to competently deliver this intervention.

This paper describes the process from engagement to competency of ASHAs as part of a larger trial, the Communication-centered Parent-mediated treatment for Autism Spectrum disorder in South Asia (COMPASS) project which is evaluating the effectiveness and cost-effectiveness of the PASS Plus intervention in New Delhi, India (ISRCTN ID: 21454676) (http://www.sangath.in/compass/). The project has received ethical clearance from the review boards at Sangath, University of Manchester and the Health Ministries Screening Committee in India. The aim of the COMPASS trial is to support the gaps in the RBSK and support the widened mandate of the ASHA's role beyond promoting child survival and providing standardized packages of care through DEICs. This paper delineates the steps in identification, recruitment, training and supervision of non-specialist frontline workers to support the scaling up of complex interventions, an important step in achieving universal health coverage of evidence-based interventions for children with disabilities.

## Methods

This work carried out in Delhi had the following steps in the process of getting frontline workers to competency:

### 1) Mapping of families of children with autism

The COMPASS trial was recruiting families from two tertiary centres in New Delhi: Maulana Azad Medical College and associated Lok Nayak Hospital (MAMC-LNH) and All India Institute of Medical Sciences (AIIMS). The team reviewed their databases of children with autism and using this information mapped districts from the 11 within New Delhi, which had the highest concentration of children with autism. These identified districts were shared with the State Program Officer (SPO) and ASHA Nodal Officer, who issued letters of instruction to the Chief Medical Officer (CMO) of the identified districts. The aim was to support allocation of trial cases within districts to resident ASHAs thus minimizing their travel time. Steps 1 and 2 were carried out simultaneously.

### 2) Engagement with the health system

Along with mapping, the team *engaged with senior personnel in the health system*, the Senior Program Officer of the Department of Health, Delhi State Health Mission (DSHM); this involved discussions on the method of remuneration that was acceptable to the health system while allowing access to the ASHA workers.

The *engagement with the ASHAs* was planned as a two-step process. The first step were in the form of group meetings in which a standard presentation comprising a video on autism was shown at the primary urban health centres (PUHC), a nodal centre for ASHA workers. This was followed by a discussion and distribution of brochures which described the project, the intervention, its aims and their possible roles in it. ASHAs attending the meetings were informed that working with this project, would be an additional responsibility that they would have to shoulder. Remuneration or incentives the project would support for the work were discussed these were approved by the Program Officer and were linked to training and supervisions attended, sessions delivered, along with a bonus for completion of predetermined sessions per case. The ASHA supervisors, the Auxiliary Nurse Midwifes (ANMs) and Medical Officer In-charges (MOICs) were also invited to attend these engagement meetings. Key demographic data on all ASHA attendees was documented including their willingness to be contacted by the team again. The second step was a follow-up call within a week with those ASHAs who had expressed an interest during the meetings. The names of ASHAs who exhibited interest were then discussed with their respective MOICs, in order to gauge whether the ASHAs had a record of commitment and capability for additional work during the project period.

### 3) Recruitment of frontline workers

The initial *telephonic interview* was conducted with ASHAs who had shown an interest. They were given more details including the tentative project workload, required travel and their need for digital fluency with smartphones was enquired upon. They were then invited to an *in-person interview*, comprising five components: (i) An introductory presentation on the project (ii) small group reflective discussions around possible challenges for the work along with a case-based field scenario to allow the research team to gauge individual problem-solving abilities, communication, listening and collaborative skills; (iii) multiple-choice quiz to evaluate sensitivity toward disabilities and attitudes around teamwork and responsibility; (iv) digital skills task to assess proficiency with smartphones to support digital learning, data entry and navigating the basics of a smartphone interface, and finally (v) interviews to help evaluate an individual's understanding of the project activities and their commitment for long-term engagement with the project. A key inquiry was to understand the ASHA's ability to self-reflect on their personal strengths and weaknesses, to gauge the level of support from their family for this extra work and their willingness to travel out of their catchment area, if required (see Supplementary materials, Section I).

### 4) Training

Shortlisted candidates received a *seven-day training* of the PASS Plus intervention which included an overview of social communication, typical developmental parent—child interactions and foundational counseling skills. The trainings were scheduled on the weekends to facilitate ease of attendance and each training day ran for ~6 h. Training was conducted by a team of intervention coordinators (ICs) with Masters' degree in the field of psychology, early childhood education, special education, and social work (LN, PS, SL, SGR, ZA, SM); who had achieved competency in the delivery of the PASS Plus intervention under the guidance of regional master trainers (VV and GD) (26). The training focused on autism, key skills and strategies of Stage 1 of the PASS communication intervention. The training schedule with topic areas covered and average time spent on each, is included as Supplementary materials, Section II. The training aimed at supporting digital literacy, an essential component for the video –feedback methodology used in the intervention. It included a blended approach with audio-visual content on a learning management system as building blocks for discussions and role plays. Focused observation of video clips of parent child interactions; a key skill for the non-specialists was a significant part of the training. Training was participatory to accommodate the needs of different learners and the content, methods and pace was continuously adapted as per the daily feedback given by the trainees, who had no previous training in developmental disorders. Each trainee was evaluated in several ways; a pre- and post-training knowledge test, module-specific quizzes and a trainer's checklist to evaluate participation and skills (on elements such as attention, oral participation, self-reflection, punctuality, role-play performance, particularly for their ability to describe video clips chosen) during the training. The cumulative scores on these various measures, helped identify high performing ASHAs in the training. See Supplementary materials, Section III, for details on some of the assessment measures used in training.

ASHAs who performed well across these multiple indicators were selected to enter an *internship* period, in which they were assigned two families of children with autism as practice cases. An Initial Home Visit facilitated by their ICs, served to introduce the “ASHA counselor” to the family. Each ASHA was initially paired with a project-based salaried non-specialist worker, who supported the session delivery by video recording the complete session. During this period, a supportive supervision structure was established, which included individual supervision of a minimum of three independently delivered sessions to practice cases along with attendance at four group supervision sessions of peer counselors. A critical aspect of all supervision sessions was the use of a quality rating measure (see Supplementary materials, Section IV). This measure evaluates the generic counseling skills and intervention-specific skills demonstrated within a session and is adapted and refined from the quality rating measure used in previous trials (26). In individual supervisions, ASHAs received one-on-one feedback from their assigned IC and in group supervision the peers were also encouraged to give feedback under guidance, so as to collectively develop peer supervision skills. Based on the skill or knowledge deficit mapped on the quality rating measures, group refresher trainings were held to build skills and team cohesion. ASHAs were encouraged to additionally fill a short self-reflection questionnaire which supported them to critically evaluate sessions they themselves had delivered.

### 5) Competency assessment

After delivering a minimum of three sessions across two practice families and if considered prepared on supervisory feedback, the counselor undertook a competency assessment. The PASS competency assessment is an objective review of both the knowledge and skills of a non-specialist. The former is assessed via multiple choice questions (e.g., on autism, communication and on case-based scenarios) and the latter was designed to be evaluated through an objective role-play of standardized session scenarios. The competency measure scores both general counseling skills (for e.g., the use of open-ended questions, paraphrasing and validating) as well as intervention-specific skills (such as identifying relevant video clips, choosing the correct strategy and addressing parent concerns). All trainees were unfamiliar with role-plays as an evaluation measure which resulted in some of them under performing during this component. To support a more realistic assessment of their skills an additional element was added to the competency measure, where an independent IC rated a recorded practice session using the same rating scale. ASHA counselor who were unable to achieve competency continued within their internship sessions before they were allowed to attempt the assessment again.

## Results

We describe the results of the steps followed to support a disability naïve frontline worker from the health system in New Delhi, India, called an ASHA, to gain skills to become competent in the delivery of a complex social communication intervention for autism; PASS Plus.

### 1) Mapping of families of children with autism

Forty three PUHCs in eight districts were identified at the time of mapping, and the project was given permission to access 26, however only 23 of these across seven districts, were approached, based on health systems recommendation and the concentration of mapped families.

### 2) Engagement with the health system

Besides access to engage with 26 PUHCs in seven of the 11 districts of Delhi, the project received permission to incentivize the ASHAs for the training, supervision and sessions delivered for the project. The amount was agreed upon by the State Program Officer DSHM which reflected a pragmatic amount that the health system could support for scale up.

The *engagement meetings with frontline workers* took place in two tranches; from February 2019 to April 2019 and from April 2019 to May 2019. In some PUHCs, the MOICs and ANMs also attended the meetings. In the first tranche, the team visited 14 PUHCs across the South, Southeast, East, and Central districts. Two PUHC's had a high percentage of newly recruited ASHAs and were not approached. In the second tranche, nine PUHCs across Northwest, West, East and Southwest districts were visited. Renovations in one PUHC of the Southwest district prevented a visit. MOICs of most PUHCs ensured maximum attendance at these engagement meetings. A total 408 of registered 466 ASHAs attended these group meetings. These ASHAs (*n* = 408) represented districts which are tabulated in Table 1.

**Table 1:** Summary of PUHCs in Delhi and those approached by COMPASS.

**S.No**.	**District name**	**No. of PUHCs permitted to access**	**No. of PUHCs approached**	**Total no. of ASHAs attending/** **capacity** ^*^
1.	SOUTH	3	3	34 (36)
2.	SOUTH EAST	2	2	51 (55)
3.	CENTRAL	7	5	62 (70)
4.	EAST	5	5	91 (106)
5.	NORTH WEST	3	3	72 (84)
6.	SOUTH WEST	3	2	66 (74)
7.	WEST	3	3	32 (41)
**Total**		26	23	408 (466)

* *Total ASHAs registered at the given Primary Urban Health Centers*.

Of the 408 ASHAs at the engagement meetings, 188 ASHAs showed an interest in the project on the follow-up call; 218 ASHAs declined the invitation to join; while two left immediately after the presentation without sharing their contact information. A few ASHAs asked for more time to support discussions with family members. Reasons given by ASHAs (*n* = 218) who declined to join the project included the inability to take on additional responsibility for a multiplicity of reasons which included; their current ASHA or household workloads, having dependent young or old family members, personal health concerns, a reluctance to travel and disinterest in engaging with new digital skills. A number of them reported a lack of family support in taking on this extra work, while in some PUHCs a lack of peers engaging with the project dissuaded a few from joining. To help support the last concern, the project strategized to recruit at least two ASHAs from one PUHC, as peer support.

### 3) Recruitment of frontline workers

ASHAs who received a positive recommendation from their MOICs were prioritized for the first tranche (*n* = 125). In the second tranche we widened the circle for recruitment to include ASHAs (*n* = 47) who showed motivation on the initial telephonic outreach. We included these recommended ASHAs along with those who had shown interest at the group meetings for *telephonic interviews*. Of 172 ASHAs, 127 engaged with the telephonic interview; 41 ASHAs who declined the telephonic interview cited similar reasons as above. Some additionally stated a lack of confidence in their line managers extending them support for this extra work, others revealed that they were already engaged in other private employment to supplement their incomes. ASHAs who owned smartphones and were familiar with digital applications were given a preference (*n* = 14). This latter criterion was eased for the second tranche and instead a digital literacy module was included within the training.

Seventy two ASHAs were identified for *in-person interviews*, of which only 45 attended. We were unable to reconnect with the majority of the ASHAs (*n* = 21) who did not attend the interview, though the six who responded stated that the geographic distance from the training centre was a barrier or that they were unavailable on the day of the interview. The age range of ASHAs attending the training represented women from 31 to 55 years; their work experience ranged from 6 months to 11 years (mean of 7 years) and their educational qualifications varied from 10th grade completion (*n* = 25); high school completion (*n* = 15), one with a diploma and seven with graduate education.

Thirty three ASHAs were selected for training based on their total interview scores. Of these, 20 ASHAs attended training and the majority (*n* = 9) of those declining to attend did not provide a reason. Of the other non-participating ASHAs, two were not supported by their families to take up this additional work, one ASHA shared concerns around her health which prevented her from travel associated with this work, and one ASHA clearly stated her dissatisfaction with the value of the incentives being offered and requested a salary.

### 4) Training

Eighteen of the 20 ASHAs who attended the first day of the training, completed it. Two ASHAs didn't attend all the days of training stating health concerns. For ASHAs who missed training days due to work at the PUHC or some personal obligations over the weekends, catch-up training was organized on weekdays. Based on the knowledge and skills gained during the training, 15 ASHAs were selected to proceed into the *internship* to deliver practice sessions under supervision.

The COMPASS team also obtained the functionality scores for the previous 6 months for the ASHAs. These are scores are tracked by the DSHM against the core activities that ASHAs need to conduct every month (e.g., immunization coverage, registering and supporting pregnancies, percentage institutional deliveries). ASHAs need to achieve a minimum of 50% on these core activities which allows their individual performance to be incentivized. An ASHA is deemed “functional” if they achieve a minimum of fifty percent every month. All ASHAs shortlisted for COMPASS had achieved this.

Six of the 15 ASHAs disengaged with the project during the internship period. One resigned from her duties in the health system, three ASHAs refused to join since their peers were not shortlisted, one ASHA found it difficult to balance both the project and ASHA workloads and another started experiencing significant health issues.

### 5) Competency assessment

Seven of the nine ASHAs passed the objective competency assessment. Collins et al. presented 24 shared “core” competencies for all mental, neurological, and substance use disorder providers that included empathic communication (27). Of these 24 competencies, there are six around screening and identification, two around formal diagnosis and referral and 16 around treatment and care. Of these, the training process was able to support the development of two within screening and identification (including the ability to describe the signs and symptoms of autism, and demonstrate cultural competence by an increased understanding of diverse families beliefs regarding autism); one supporting the recognition of the thresholds for referrals; and seven from the treatment domain (including supporting a community-based intervention for young children with autism, supporting the mental health of parents, developing and establishing a therapeutic alliance with the family). These competencies were measured during the assessment conducted after each counselor had delivered a minimum of three sessions across two practice families.

The 1998 National Community Health Advisor Study, conducted in the United States, identified a list of 18 qualities that are essential to have as a successful community health worker (28). Of these “being connected to the community” is a key characteristic of the ASHA defined by her being a resident in the community that she works in. As a trusted member of the local community who already conducts home visits, this made her the ideal choice for our intervention delivery. Other qualities such as being open-minded, non-judgmental, motivated and capable of self-directed work, and empathy (28), were evaluated through our interviews and training process, wherein we found that those ASHAs who across the interview phase and during their training and internship, could openly talk about their challenges and were observed to be motivated, committed, and showed openness to learn novel things managed to make their way to competency (see Supplementary materials, Section I).

Even after 9 months of supportive supervision and multiple refresher trainings, two ASHAs were unable to gain optimal skills during their internship and were discontinued from the project. The mean time to competency was just over 8 months, but was impacted by a number of the rate limiting issues which included the delay in assigning practice cases, and personal reasons which caused certain trainees to take a temporary break from the project.

**Figure 1:**
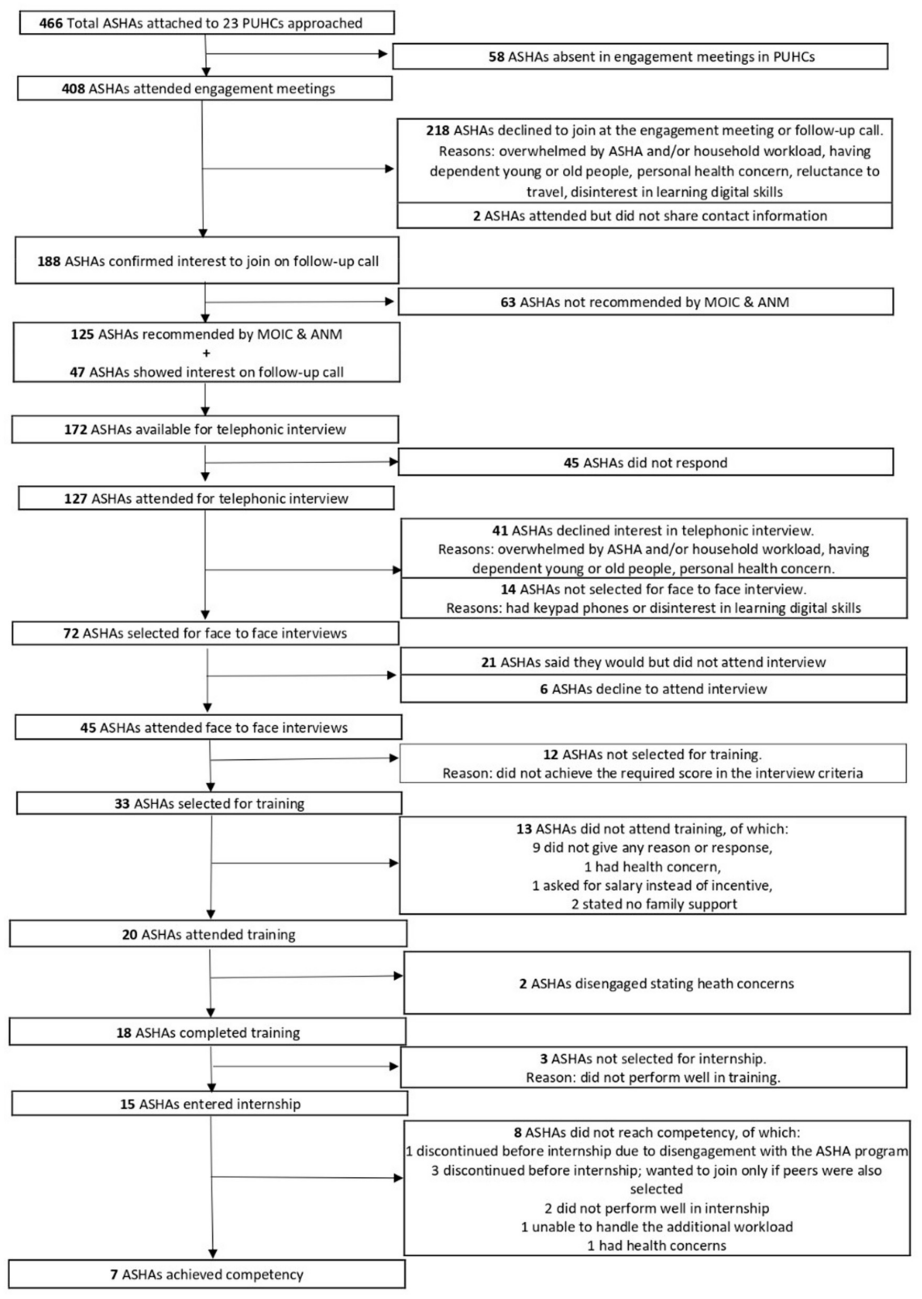
Flowchart depicting number of ASHAs involved at various stages of the project.

The mean age and work experience of seven ASHAs who achieved competency is similar to that of all ASHAs (*n* = 45) interviewed. However, the overall gain in knowledge from the training of 7 competent ASHAs averaged (mean percentage scores 64% pre-training and 81% post-training scores) higher than that of 13 ASHAs who did not achieve competency (mean percentage scores 47% pre-training and 65% post-training). ASHAs who were above 50 years of age and had been in service for 10 or more years were minimally represented in the group chosen for training, and those that did, did not achieve competency. Figure 1 illustrates the attrition of ASHAs across the various stages of involvement with the project.

## Discussion

We set out to evaluate whether health system frontline workers, the Accredited Social Health Activist, could be supported to learn and deliver a complex social communication intervention for autism in New Delhi, India. The process described here is nested in a larger ongoing effectiveness and cost-effectiveness trial and the impact of the ASHA training on intervention delivery to families with an autistic child will be published when the trial is complete. There is now substantial evidence for the potential of non-specialist frontline workers to effectively deliver a wide range of preventive, promotive and curative services (29). It has been shown that they can play a role in meeting the needs of poor and marginalized populations in contexts of weak health systems, resource constraints, and vast inequalities. In urban populations, frontline workers perform important roles that other providers are not well-positioned to deliver, including roles related to outreach, behavioral change, psychosocial support and managing chronic diseases (30). With this background knowledge, PASS Plus has been designed to address challenges faced by families of children with autism and is a community-based non-specialist delivered intervention. In the context of India, ASHAs are mandated to conduct home visits for improving the health within communities (31) and their core counseling skills were identified as a suitable choice for delivering a psychosocial intervention for autism, with a view to the potential for scaling up an evidence-based intervention.

In the current study, though we aimed to train a larger number of ASHAs, we were only able to get a fraction of the initial attendees interested in the work. There are a number of reasons for this. The initial high attrition can be attributed to the hierarchical system that ASHAs work in; this meant that many who may have been disinterested in the work were expected to attend the engagement meetings. Since the majority of ASHAs were naïve to disabilities, we wanted the process to support the identification of those individuals who were able to commit to additional work beyond their mandated activities. Our process revealed some obvious but also interesting characteristics. We found that years of work experience was not an accurate marker for engagement with this project, instead it was an inclination to learn something novel and to engage in new tasks which were critical to success. We noted that ASHAs who were in the position for many years seemed to lack the motivation to take on new challenges. On the other hand, new recruits to the health system, were still learning their core curriculum and were unable to take on the additional workload. In addition, the functionality scores we had obtained from the DSHM were neither indicative nor predictive of an ASHA's ability to deliver this complex intervention.

An ASHA's work is a part-time voluntary activity for which she receives an honorarium, incentivized for activities delivered. As a result, we found that an ASHA whose own personal commitments were significant [e.g., those with young children, or elderly dependents], were unable to make the time for this project. Over the period of internship, it was the ASHAs who had agency, reflected in their ability to make informed independent decisions without consulting other household members and who demonstrated the confidence to travel out of their catchment area, were able to engage with this work.

The PASS Plus intervention uses the technique of video feedback, and hence requires a level of digital literacy for its delivery. This influenced an individual's ability to be trained and succeed in delivering this intervention. Our finding that even without access to a smartphone, individuals who were keen to learn were able to enhance their personal digital literacy under supervision. It was the ASHAs who asked questions, repeatedly tried to master practical tasks, and who engaged with the topics being discussed, that achieved proficiency in the reflective approach used during session delivery for PASS Plus. Our training and supervision processes supported the development of ten of the 24 competencies suggested by Collins et al. (27), however, further research should aim to track quality of these competencies over time when such a program is implemented at scale.

A critical aspect of the project which has allowed the ASHAs we trained to deliver a quality intervention has been the building of a framework of supportive supervision and developing a methodology to encourage peer-to-peer support during the internship period. This helped create a skilled set of workers who have been able to stay motivated, engaged and keen to improve their skills. An objective quality rating measure and self-reflection questionnaire used consistently across sessions, allowed trainees to gain skills in evaluating sessions effectively. We observed that those who demonstrated the capacity of accurate self-reflection for their own sessions and had the ability to receive constructive criticism from peers and supervisors were able to quickly move to competency. The involvement of peers in programmatic training and supervision will be critical to ensuring the scalability of task-sharing for complex interventions in the future. However, these observations need to be studied systematically through further research.

A key limitation of our study is that we only worked in an urban setting of Delhi and hence the workers themselves may not be representative of non-specialist frontline workers across the country. Additionally, we were only able to reach out to a small number of ASHAs since this work was conducted within the timelines of the research project. It is possible that from a larger group we may have been able to identify a higher percentage of individuals who could be supported to gain competency.

Future empirical studies should also objectively measure the various personal attributes of community health workers such as the 18 qualities described by Brown et al. (28) along with those revealed through our processes, which may support the achievement of competency in delivering such complex intervention, and could be compared across intervention studies. The WHO Global Recommendation and Guidelines on task-shifting (32) states that having clear recruitment criteria that states behavioral and technical skills needed for the job makes identification of desired qualities in a candidate easier. Within India itself, there are other frontline workers such as an Anganwadi Teacher (AWT) or ANMs who may also be appropriate to deliver this intervention, depending on the context of the community, but this exploration was beyond the scope of this study.

With the prevalence of autism at one percent (5); it is clear that we do not need to train every ASHA in the intervention, however there is a need to be able to identify individuals who could potentially support families for any program that aims to scale up intervention services for children with autism. Through the methodology we used, we were able to identify key characteristics that would help the health system identify ASHAs who would be able to deliver complex interventions for autism.

Through this systematic process of recruitment, training and supportive supervision we have demonstrated a feasible methodology of training non-specialist frontline workers with no exposure to autism, to achieve competency in delivering a complex intervention using digital technology. However, we recognize that the process of identification of the characteristics of workers who could take on these roles needs refinement to optimize the effort made to achieve this at scale. While we await effectiveness data, this process illustrates a possible approach of scaling up an evidence-based intervention delivered within a supervisory framework. From expensive, difficult to reach city-based centers to community-based delivery in the homes of families of children with disabilities. This is an early indication that we can envision a system of support for vulnerable families of children with complex neurodevelopmental disorders. This support entails high quality services within their communities by a disability-specific worker, who can be trained across a number of disability modules and could then be integrated within the existing health system frameworks such as the RBSK in India.

## Data availability statement

The original contributions presented in the study are included in the article/Supplementary material, further inquiries can be directed to the corresponding author/s.

## Ethics statement

The studies involving human participants were reviewed and approved by Sangath IRB, Indian Council of Medical Research and the University of Manchester's Ethics Committee (UREC 2; ref no.: 2019-5223-11996). Written informed consent for participation was not required for this study in accordance with the national legislation and the institutional requirements.

## Author contributions

GD, VV, CT, RR, VP, and JG conceived the idea for the research paper. LN, PS, SM, SR, SL, and ZA conducted literature review, with support from GD. RR, DK, and MA were involved in the formative work and engagement with the health system. ZA, SR, SM, PS, and LN were involved in mobilizing engagement meetings. LN, PS, SM, SR, SL, and ZA were involved in processes of recruitment, training, internship, supervision, and competency assessment of ASHAs. LN, PS, SM, ZA, SR, SL, and GD drafted the first version of the manuscript. LN, PS, SR, RR, VV, CT, VP, JG, and GD contributed to subsequent versions. All authors have read and approved the final manuscript.

## Funding

This work has been conducted with the support of a Medical Research Council, UK grant. Grant Reference MR/R006164/1.

## Conflict of interest

The authors declare that the research was conducted in the absence of any commercial or financial relationships that could be construed as a potential conflict of interest.

## Publisher's note

All claims expressed in this article are solely those of the authors and do not necessarily represent those of their affiliated organizations, or those of the publisher, the editors and the reviewers. Any product that may be evaluated in this article, or claim that may be made by its manufacturer, is not guaranteed or endorsed by the publisher.
