# Supplementary material for: From engagement to competency: The pathway to making disability naïve frontline workers competent in the delivery of an evidence-based autism intervention in New Delhi, India
Source: Front Psychiatry. 2022 Jul 29;13:903341. doi: 10.3389/fpsyt.2022.903341 (PMC9372267; doi:10.3389/fpsyt.2022.903341)

### SUPPLEMENTARY MATERIALS

### Section I: ASHA Recruitment measures

### Summative scoring sheet

| **CANDIDATE ASSESSMENT SHEET** | |
| --- | --- |
| Position**:** ASHA COUNSELLOR | |
| **CANDIDATE NAME** |  |
| Academic Qualification | Free text |
| Level of Education | Free text |
| Previous Experience (years in service) | Free text |
| **GENERAL ENGAGEMENT (12)** | |
| Please rate the candidate on a scale of 1 to 3 (1 is for poor, 2 is for average, 3 is for good) | |
| Stayed relevant to the topic of discussion |  |
| Engagement in group work |  |
| Willingness to take initiative (to scribe and to present) |  |
| Problem solving skills |  |
| **DIGITAL SKILLS (6)** | |
| Please rate the candidate on a scale of 0 to 2 (0 is for couldn’t complete the task/asked for help; 1 is for completing but took longer time than specified; 2 is for completing the task quickly) | |
| Unlock the phone with password |  |
| Switch on WIFI and Open a Hindi website |  |
| Type using Hindi keypad |  |
| **FACE TO FACE INTERVIEW (12)** | |
| Please rate the candidate on a scale of 0 to 2 (0 is for poor; 1 is for average, 2 is for good; give 0.5 for score that fall between these) | |
| Understanding about the role |  |
| Key responsibilities (training, session delivery and equipment) |  |
| Sensitivity to children with disability (why they want to work on this project) |  |
| Ability to talk about challenges comfortably (experience in the  field or in training as an ASHA) |  |
| Free time in a week/ Ability to work on weekends |  |
| What is your biggest quality/strength? | Free text |
| If you could change something about yourself (a habit or quality) what would it be? | Free text |
| What is your preferred style of learning? | Free text |
| Please rate the candidate (0 is for no; 1 is for yes) | |
| Can independently go for sessions |  |
| Punctuality (from attendance sheet) |  |

| MCQ (5) | |
| --- | --- |
| Score on MCQs |  |
| **GRAND TOTAL (35)** |  |

| Decision Taken |  | Recommend |
| --- | --- | --- |
|  |  | Not Recommend |
|  |  | Hold |
| RANK |  | |
| **Final remarks of the interviewer:** | | |
| Name: | | |
| Signature: | | |
| Date: | | |
| Designation: | | |

# General group engagement (Small group activity)

**INSTRUCTIONS**

Each participant calls out the count of 1 and 2 to divide themselves in two groups. Each group has one IC who facilitates the discussion. Each group is informed that they have to note down the points discussed on a flip chart and then present their ideas to the other group. The group chooses who will scribe and who will present. The IC facilitates gives them the option of scribing and presenting the way the group wants and give them 2 minutes for deciding it. ICs rates the participants in their group (7 each) as per the criteria mentioned below, a third IC floats among both group and rates all the participants and collates later. Audio record the group discussions.

# QUESTIONS

- What they think they could gain from this experience?
- What they think can be the potential challenges for them?
- What are the prevalent views and opinions of mental health and illness in our communities?
- Suppose a family with a pregnant woman have recently moved from a village. The woman insists on having her baby delivered at home. She is scared of hospitals and feels that home delivery is the best for the child as her first-born was delivered at home. How will you get her to deliver at the hospital? (For problem-solving)

# SCORING CRITERIA

1. stayed relevant to the topic of discussion

| Score 1 | If says things irrelevant to the topic |
| --- | --- |
| Score 2 | If says things in line with the topic but deviates sometimes |
| Score 3 | If says relevant things in line with the topic |

1. Engagement in Group work

| Score 1 | If stays passive in the group discussion mostly, even when given a clear chance to speak. |
| --- | --- |
| Score 2 | Speaks only when given a chance to speak and if adds to the point others make |
| Score 3 | States opinions clearly and also gives others a chance to speak |

1. Willingness to take Initiative

| Score 1 | Doesn’t take initiative, remains passive, and turns down the opportunity to write or present even asked to. |
| --- | --- |
| Score 2 | Seems willing to scribe or present, give others a chance to present |
| Score 3 | Takes clear initiative to scribe or present; builds a consensus with the group to scribe or present |

1. Situation based problem solving skills

| Score 1 | Says a few words only when a probed, or says only that it’s the right thing to do. |
| --- | --- |
| Score 2 | Says we’ll make them understand, mention a few points of how; adds to the point others make |
| Score 3 | Brings up points like pros and cons of both the situations in details of both |

# C) Digital tasks instructions

**DIGITAL WORK INSTRUCTION SHEET** **(Total Time: 20 Minutes)**

Direction**s**: Three tasks have been given in this paper. Before starting the work, read this letter carefully for 5 minutes. When each task is completed, raise your hand and notify the supervisor. Only after that start other tasks.

**Task 1: Unlock the phone/tab with the given password (Time: 5 Minutes)**

• Turn on the phone or tab you have in front of you and then unlock it with the password below.

**Password is :__________**

**Task 2: Open website (Time: 5 minutes)**

1. Turn on Phone/Tab

2. Enter Password

3. Those who have the phone – Go to Settings -> Wireless or Networks -> ON WIFI

Those who have the tab - Go to Settings -> Connections -> ON WIFI

4. Then go to Google chrome and open the website given below in Hindi

5. Website is: - jagran.com

**Task 3: Type using Hindi keypad (Time: 5 Minutes)**

*A) For those who have a phone –*

1. Open Notepad on the phone

2. Then at the bottom there is a + in the middle, press it

3. Then type one line from the line given below in Hindi

*B) Those who have Tab –*

1 Open Samsung Notes app in tab

2. Then on the right hand side there is a symbol “ **+ ”** , press it

3. Then type one line from the line given below in Hindi

**The line is :-**

I. Once a crow was very thirsty. He was not getting water anywhere.

OR

II. There was very little water in the pot. The crow's beak could not reach the water.

### Multiple choice questions

Q1. What is a good thing to do if a child comes to you with their problem?

1. Tell what they must do
2. Tell them to obey their parents
3. Listen and try to understand what they are trying to tell you
4. Tell them you don’t have time

Q2**.** The best way to get someone to take advice from you is to

1. Insist that they follow what you are telling them
2. Tell that they can do what they like
3. Allow them to discuss their views on what you have told them
4. None of the above

Q3. You are working as a COMPASS counsellor and you are unable to make an appointment with a family. What would be the best option?

1. Immediately call the family, tell them the reason and set up a new appointment
2. Cancel the appointment
3. Immediately call the family, tell them the reason and set up a new appointment and inform your supervisor
4. Send another counsellor to speak to the family

Q4. A team works well if all members share

1. The same final goal
2. The same values
3. Good communication skills
4. All of the above

Q5. If a family member gets very upset and cries in front of you, what would be the best response?

1. Leave the room and allow them to continue
2. Tell them to stop crying and get on with their work
3. Go out and come back when they stop crying
4. Gently ask them what they are upset about, listen and give your opinion if you think it will help

# One-on-one interview questions

- 1. **Understanding the role**
     1. If you reflect on the presentation showed to you in the morning, what all work do you think you will be doing as part of being a COMPASS counsellor?
     2. Do you remember which disability in children are we working with in COMPASS project?
     3. What are some of things you will have to do in a session?

## Key responsibilities (training, session delivery and equipment)

- - 1. How will you take care of the equipment given to you?
    2. How will you be responsible for the session delivery?
    3. How will you be responsible for your training?

## Sensitivity to children with disability

- - 1. Why do you want to work with these families of children with autism?

## Ability to talk about challenges comfortably

- - 1. In your experience of working as an ASHA in the field or during training what were there challenges?

## Free time in a week/ Ability to work on weekends

- - 1. How much free time do you have in a week? How you spend your weekends, how comfortable are you working on weekends.

### Section II: 7-days Training Schedule

| **TRAINING TOPICS** | **DAY** | **AVERAGE DURATION** |
| --- | --- | --- |
| Introduction to Sangath | 1 | 0.30 hrs |
| Introduction to Assessments | 1 | 0.25 hrs |
| Communication | 1 | 1.25 hrs |
| Parent-Child Interaction | 1 | 0.5 hrs |
| General Counselling Skills | 2 | 1.5hrs |
| Introduction to Autism | 2 | 2.5 hrs |
| Explaining PASS PLUS | 2 | 0.5 hrs |
| Structure of PASS Plus Session | 2 | 1 hr |
| PASS Plus Specific Counselling Skills | 3 | 1 hr |
| Stage 1 | 3 | 1.5 hrs |
| Stage 1: Strategy 1 | 4 | 4.5 hrs |
| Stage 1 : Strategies 2 & 3 | 5 | 4.5 hrs |
| Stage 1: Strategies 4 to 6 | 6 | 4.5 hrs |
| Initial Home Visit, Home program, Competency, Quality measure | 7 | 2 hours |

### Section III: Daily in-training assessment of trainee’s sheet

## Assessment of Trainees (Day 1-4)

Name of Trainer: Date:

Rating Scale; 1 = Behaviour Seen; 0 = Behaviour Not Seen

NOTE: Score 0 if the behaviour was not seen on any instance, if the behaviour was present at all during the interaction give a sore of 1 – please give examples.

Score each trainee on the following

| Name of the trainee | Attention /Interest | Oral Participation | Self-Reflection  (Refer to trainees feedback form) | Punctuality | Video Description | Total  Score  (5) | Remarks/ Justification |
| --- | --- | --- | --- | --- | --- | --- | --- |
|  |  |  |  |  |  |  |  |
|  |  |  |  |  |  |  |  |

## Assessment of Trainees (Day 5 & 6)

Name of Trainer: Date:

Rating Scale; 1 = Behaviour Seen; 0 = Behaviour Not Seen

NOTE: Score 0 if the behaviour was not seen on any instance, if the behaviour was present at all during the interaction give a sore of 1 – please give examples.

Score each trainee on the following

| Name of the trainee | Attention /Interest | Oral Participation | Self-Reflection  (Refer to trainees feedback form) | Punctuality | Video Description | Choosing relevant clip | Role play performance | Total  Score (7) | Remark/ Justification |
| --- | --- | --- | --- | --- | --- | --- | --- | --- | --- |
|  |  |  |  |  |  |  |  |  |  |
|  |  |  |  |  |  |  |  |  |  |

## Assessment of Trainees (Day 7)

Name of Trainer: Date:

Rating Scale; 1 = Behaviour Seen; 0 = Behaviour Not Seen

NOTE: Score 0 if the behaviour was not seen on any instance, if the behaviour was present at all during the interaction give a sore of 1 – please give examples.

Score each trainee on the following

| Name of the trainee | Attention /Interest | Oral Participation | Self-Reflection  (Refer to trainees feedback form) | Punctuality | Total  Score (4) | Remarks/ Justification |
| --- | --- | --- | --- | --- | --- | --- |
|  |  |  |  |  |  |  |
|  |  |  |  |  |  |  |

### Section IV: Quality Assurance measure for sessions


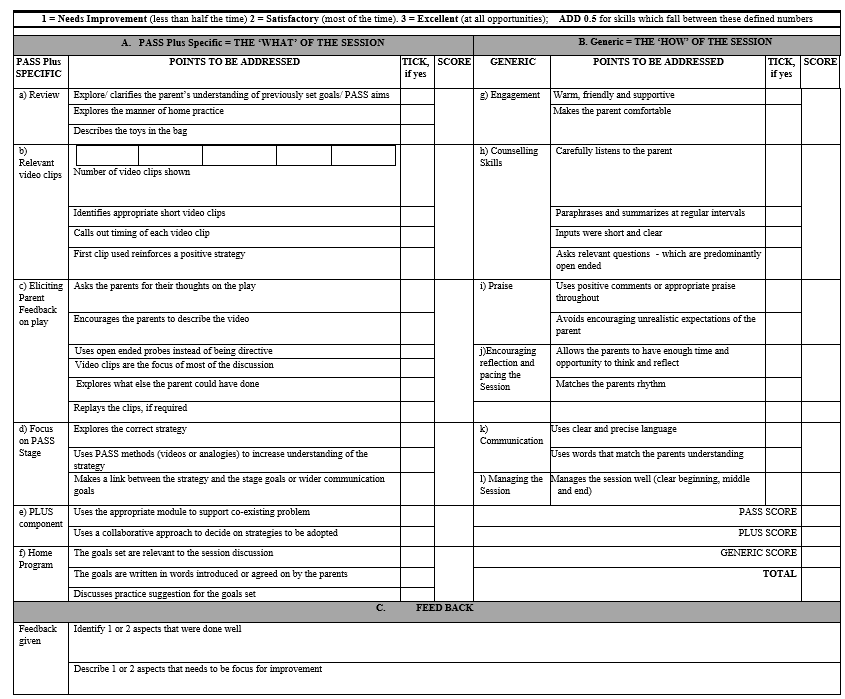

Supplement: Supplementary file 1 [file Data_Sheet_1.docx]
